# Social determinants of antenatal depression and anxiety among women in South Asia: A systematic review & meta-analysis

**DOI:** 10.1371/journal.pone.0263760

**Published:** 2022-02-09

**Authors:** Nafisa Insan, Anthony Weke, Simon Forrest, Judith Rankin

**Affiliations:** 1 Population Health Sciences Institute, Newcastle University, Newcastle Upon Tyne, United Kingdom; 2 Department of Sociology, Durham University, Durham, United Kingdom; University of Gondar, ETHIOPIA

## Abstract

**Background:**

Pregnancy is a time of major psychological changes making pregnant women more susceptible to depression and anxiety. Prevalence is higher among women living in Bangladesh, India and Pakistan, compared to high-income countries, due to poor understanding and lack of mental health integration within antenatal care. Antenatal depression/anxiety is associated with adverse outcomes including postnatal depression, low birth weight and impaired fetal development. Existing systematic reviews provided only limited information on the social determinants of antenatal depression/anxiety in these South Asian countries.

**Objective:**

This review aimed to identify, synthesise and appraise the evidence on the social determinants associated with antenatal depression and anxiety in women living in Bangladesh, India and Pakistan.

**Methods:**

We searched five databases (MEDLINE, Embase, PsycINFO, Scopus, Web of Science) and PROSPERO. Observational studies published between 1^st^ January 2000 and 4^th^ January 2021 were included if they were in the English language, used validated tools for measuring depression/anxiety in pregnant women and reported statistical associations or raw numbers. Summary estimates were obtained using random-effects model. Heterogeneity and publication bias was measured using the I^2^ statistic and Egger’s test, respectively. This review was registered on PROSPERO (reference: CRD42020167903).

**Results:**

We included 34 studies (with 27,379 women). Meta-analysis of Adjusted Odds Ratios (AOR) found that Intimate partner violence (AOR 2.48, 95% CI 1.41–4.33), unplanned pregnancy (AOR 1.53, 95% CI 1.28–1.83), male gender preference (AOR 3.06, 95% CI 1.40–6.72) and poor relationship with in-laws (AOR 2.69, 95% CI 1.25–5.80) were significantly associated with antenatal depression/anxiety.

**Conclusion:**

The review identified a complex range of social determinants of antenatal depression and anxiety in Bangladesh, India and Pakistan. Screening tools to identify pregnant women at high risk should be integrated within antenatal care to prevent adverse outcomes. Knowledge of these social determinants will inform the development of such screening tools and interventions.

## Introduction

Pregnancy is a time of major physiological, hormonal, and psychological changes placing women at an increased risk of emotional changes during and immediately after pregnancy [1]. Maternal mental health is recognised as a worldwide public health issue, with approximately 10% of pregnant women and 13% of women who have just given birth experiencing some sort of mental health condition, particularly depression and comorbid anxiety [2]. Evidence suggests that two thirds of cases of postnatal depression actually present antenatally, before childbirth [3, 4], making the antenatal period a key stage to target in interventions. Depression and anxiety during pregnancy results in adverse outcomes in the mother and child including increased risk of postnatal depression, low birth weight and impaired fetal development [5].

Prevalence of antenatal depression ranges from 7 to 15% in high-income countries (HICs) and 19 to 25% in Low- and Middle-Income Countries (LMICs) [6]. These disparities result from the lack of mental healthcare integration into antenatal care, poor understanding and acceptance of mental health conditions and higher socioeconomic deprivation in LMICs [7]. It is likely that these reported percentages in LMICs are underestimates due to the stigma attached to mental health preventing people from understanding, acknowledging and sharing these issues within these populations [8]. This is particularly prominent among women who are often victims of gender-based violence, experience lack of family planning and low support leading to depression during pregnancy [7]. These are commonly reported social determinants of maternal depression [7, 9]. Social determinants are “the conditions in which people are born, grow, live, work and age”. They are crucial to improving health since they are “mostly responsible for health inequalities” [10].

Bangladesh, India and Pakistan are LMICs which form a major part of the Indian subcontinent of South Asia. Although there is heterogeneity within and between these countries, they share cultural and social similarities which are reflected in a degree of commonality in the challenges and social inequalities faced by women living in these countries. For this reason, they are often studied together to enhance understanding of health phenomena within South Asia [11].

A longitudinal study carried out in 2008–2009 in Bangladesh found the prevalence of antenatal depression was 18% and antenatal anxiety was 29.4% [9]. Studies in India have reported a wide-ranging prevalence of antenatal depression between 9.8 and 36.7% [12–14] and a prevalence of 55.7% for antenatal anxiety [15]. In Pakistan, the prevalence of antenatal depression ranges from 18% [16] to 39.5% [17] and the prevalence of antenatal anxiety is approximately 34.5% [18]. The varying prevalence rates found between and within these countries may be a result of methodological differences in sample size, sample characteristics and region of study. It may also highlight the heterogeneity between the countries.

Existing systematic reviews investigating risk factors of antenatal depression/anxiety [6, 19, 20] do not include meta-analyses to determine pooled summary estimates of association. Only two reviews included any studies in Bangladesh, India or Pakistan [6, 20], however, they did not capture the range of studies published in these countries and only included papers published to 2015. To our knowledge, there are no known systematic reviews and meta-analyses investigating the social determinants of antenatal depression in Bangladesh, Indian and Pakistan where women face increased vulnerability to mental health issues and social inequalities. Understanding social determinants of antenatal depression and anxiety will enable holistic care for pregnant women in these countries where mental health is given less attention [21]. This understanding will assist in the development and implementation of tailored interventions and programmes for pregnant women to reduce symptoms of depression and anxiety during pregnancy and, consequently, positively impacting health after childbirth. This study aimed to identify, synthesise and appraise the available evidence on social determinants of antenatal depression and anxiety in women living in Bangladesh, India and Pakistan, and to identify which determinants have the most impact on antenatal depression and anxiety.

## Methods

Methods and reporting were conducted in accordance with the Meta-analysis Of Observational Studies in Epidemiology (MOOSE) guidelines (S1 Table). Observational studies investigating social determinants of antenatal depression or anxiety in any region of Bangladesh, India and Pakistan published in, or translated into, the English language since 1^st^ January 2000 to 4^th^ January 2021 were included in this review. To be included, studies had to use validated tools for measuring antenatal depression/anxiety and either reported the odds ratios (OR) and 95% Confidence Intervals (95% CI) or provide sufficient raw data for ORs to be calculated. Randomised controlled trials and studies using only a specific sub-group of pregnant women were excluded.

A systematic literature search was conducted via the electronic databases MEDLINE, Embase, PsycINFO, Scopus, Web of science using keywords and MeSH headings developed with an information specialist in accordance with the PICOS framework (see S1 Fig for full search strategy). PROSPERO was also searched to identify if there were any similar systematic reviews conducted already. Search results from the electronic databases were exported into the reference manager Zotero and screened independently by two investigators. Zotero provides a platform to export studies from databases, automatically remove duplicates and to conduct screening of titles. Where there were disagreements between the two reviewers regarding eligibility, discussions would occur to resolve it, or a third reviewer was included, however, this was not required in this review. Grey literature was searched using the Open Grey database and also via charities and non-governmental organisations such as Building Resources Across Communities (BRAC), UNICEF, National Health Mission India and International Centre for Diarrhoeal Disease Research Bangladesh. Supplementary searches included hand searching study reference lists included from databases and citation searches using Google Scholar. Authors were contacted when full texts of studies were not found, or additional data were required for inclusion in the meta-analysis. Database searches were completed on 31^st^ January 2020 and updated on 4^th^ January 2021.

Data extraction was carried out independently by two researchers using a standardised protocol (S2 Table). The Newcastle-Ottawa Scale adapted for cross-sectional studies which assesses information bias, selection bias and confounding bias (S3 Table; S2 Fig) was used to assess study quality.

### Data synthesis and statistical analysis

Data synthesis was grouped by social determinants. Only those social determinants that were investigated by three or more studies were included in the review as fewer than three was considered as insufficient evidence for narrative synthesis, as recommended by previous systematic reviews [19, 22]. A supplementary table (S4 Table) reports the results for social determinants investigated by only one study. Although it would be preferable to investigate depression and anxiety individually, they were grouped together under one outcome in the studies. As they are comorbid common mental disorders and there was only one included study with anxiety as a separate outcome, we combined the outcomes depression and anxiety. This approach has been practiced in previous studies [23, 24]. We considered this to be the most appropriate outcome for this synthesis given the number of studies.

For determinants where pooling of the ORs were appropriate, meta-analyses were performed using the random-effects model [25], regardless of the heterogeneity. This is a slight deviation from the protocol as after screening of full texts we expected heterogeneity between the studies, thus, opted for random-effects model from the start. Heterogeneity was measured using the I^2^ statistic (0–100%) [26], with a threshold of >75% representing considerable heterogeneity as stated in the Cochrane Handbook [27]. Unadjusted and adjusted ORs were pooled separately to account for the confounding factors and both were presented to investigate the differences (see S5 Table for factors included in the included studies adjusted models). Sensitivity analysis was performed by removing studies that were assessed to be of low quality. The results were statistically significant when two-sided p-values were less than 0.05. Publication bias was tested for using the Egger’s test (p<0.05 indicates the existence of publication bias) [28]. The statistical analyses were conducted using STATA V.16.1. The review protocol was registered on the PROSPERO database of systematic reviews (reference CRD42020167903).

## Results

**Fig 1:**
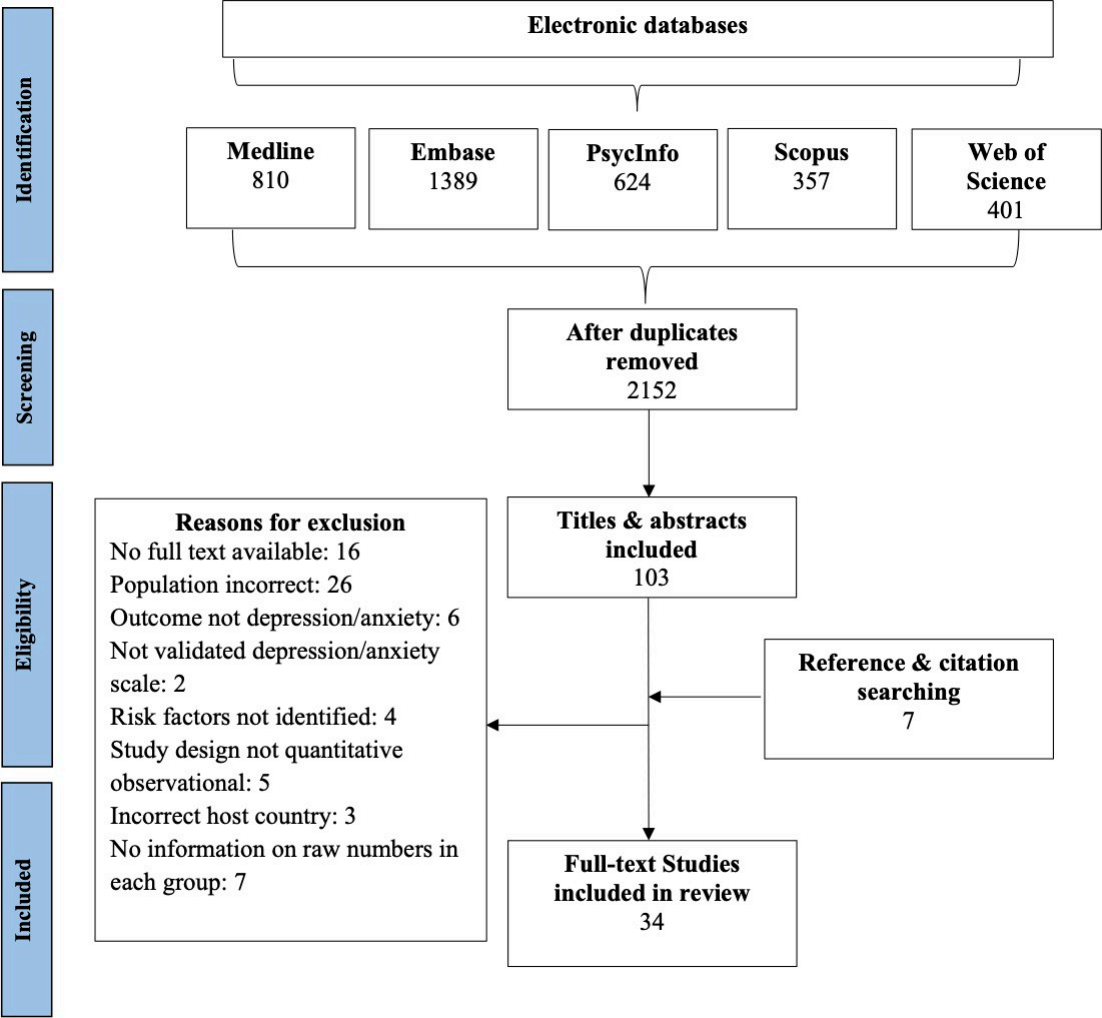
PRISMA flow diagram of included studies.

The searches identified 3,372 studies; following deduplication, 1,987 studies remained for screening. Following screening and supplementary searches, a total of 34 studies met the inclusion criteria and were included in this review (Fig 1).

The sample sizes ranged from 100 [29] to 14,629 [30] pregnant women; all the studies were cross-sectional and the majority (n = 30) have been published since 2010. Studies were predominately conducted in Pakistan (n = 20) [4, 16–18, 29, 31–45], followed by India (n = 11) [12–15, 46–52] and Bangladesh (n = 3) [9, 30, 53]. The majority of studies included samples of pregnant women recruited from hospitals (n = 24) [13–15, 18, 29, 31–38, 40–46, 48, 49, 51, 52], with the remaining being community-based samples (n = 10) [4, 9, 12, 16, 17, 30, 39, 47, 50, 53]. Most studies investigated depression only (n = 24) [4, 12–14, 17, 29–31, 35–39, 42, 43, 45–53], followed by co-morbid depression and anxiety (n = 9) [9, 16, 18, 32–34, 40, 44] and anxiety alone (n = 1) [15]. Quality scores ranged from one to ten out of a possible score of ten. Overall, 47% (n = 16) of the studies scored medium; 32% (n = 11) of the studies scored high; 21% (n = 7) of the studies scored low (Table 1, S3 Table).

**Table 1:** Descriptive summary of the 34 included studies.

Author, publication year (reference number)	Country	Sample size	Recruitment source	Outcome	Ascertainment of outcome (Cut-off score)	Quality score*
Ajinkya et al, 2013 [14]	India	185	Hospital	Depression	BDI ≥ 17	4
Ali et al, 2012 [18]	Pakistan	165	Hospital	Anxiety & Depression	HADS ≥ 8	6
Ayaz et al, 2019 [29]	Pakistan	100	Hospital	Depression	BDI ≥ 16	3
Ayyub et al, 2018 [17]	Pakistan	367	Community	Depression	Urdu EPDS ≥ 12	10
Babu et al, 2018 [47]	India	823	Community	Depression	Kannada Kessler Psychological Distress Scale (K-10) ≥ 10	8
Bavle et al, 2016 [48]	India	318	Hospital	Depression	Hindi EPDS ≥ 10	4
Dahiya et al, 2020 [52]	India	200	Hospital	Depression	EPDS	6
Din et al, 2016 [32]	Pakistan	230	Hospital	Anxiety & Depression	DASS-42	5
Gausia et al, 2009 [53]	Bangladesh	346	Community	Depression	Bangla EPDS ≥ 10	8
George et al, 2016 [12]	India	202	Community	Depression	CIS-R	10
Ghaffar et al, 2017 [33]	Pakistan	750	Hospital	Anxiety & Depression	Urdu HADS	8
Goyal et al, 2020 [49]	India	281	Hospital	Depression	DSM-5	6
Gul et al, 2017 [34]	Pakistan	500	Hospital	Anxiety	AKUADS > 19	7
				Depression		
Hegde et al, 2013 [13]	India	253	Hospital	Depression	EPDS ≥ 13	2
Humayun et al, 2013 [35]	Pakistan	506	Hospital	Depression	EPDS ≥ 10	5
Imran et al, 2010 [36]	Pakistan	213	Hospital	Depression	Urdu EPDS > 12	6
Jafri et al, 2017 [37]	Pakistan	300	Hospital	Depression	Hamilton Rating scale for Depression > 7	2
Jamal et al, 2018 [38]	Pakistan	197	Hospital	Depression	BDI ≥ 17	5
Karmaliani et al, 2009 [16]	Pakistan	1368	Community	Anxiety & Depression	AKUADS ≥ 13	6
Maselko et al, 2018 [39]	Pakistan	115	Community	Depression	PHQ-9	7
Mir et al, 2012 [31]	Pakistan	340	Hospital	Depression	AKUADS	9
Nasreen et al, 2011 [9]	Bangladesh	720	Community	Anxiety & Depression	STAI ≥ 45	10
					EPDS ≥ 10	
Nath et al, 2019 [15]	India	380	Hospital	Anxiety	Pregnancy-related thoughts (PRT) scale	8
Niaz et al, 2004 [40]	Pakistan	200	Hospital	Anxiety	HADS >7	2
				Depression		
Rabia et al, 2017 [41]	Pakistan	520	Hospital	Anxiety	HADS >8	5
				Depression		
Rahman et al, 2003 [4]	Pakistan	632	Community	Depression	SCAN	7
Sabir et al, 2019 [42]	Pakistan	450	Hospital	Depression	Goldberg’s depression scale > 21	4
Safi et al, 2013 [43]	Pakistan	300	Hospital	Depression	CES-D > 15	3
Sheeba et al, 2019 [46]	India	280	Hospital	Depression	EPDS >13	8
Shehroz et al, 2019 [44]	Pakistan	200	Hospital	Anxiety	HADS	1
				Depression		
Shidhaye et al, 2017 [50]	India	302	Community	Depression	EPDS >12	10
Srinivasan et al, 2015 [51]	India	100	Hospital	Depression	EPDS ≥ 10	6
Surkan et al, 2018 [30]	Bangladesh	14,629	Community	Depression	PHQ & CES-D	9
Zia et al, 2018 [45]	Pakistan	907	Hospital	Depression	BDI	2

*Quality assessment scores from the adapted Newcastle-Ottawa Scale reported in S3 Table

BDI = Beck Depression Inventory, HADS = Hospital Anxiety Depression Scale, EPDS = Edinburgh Postnatal Depression Scale, K-10 = Kessler Psychological Distress Scale, DASS = Depression Anxiety and Stress Scale, CIS-R = Clinical Interview Schedule Revised, DSM-5 = Diagnostic and Statistical Manual of Mental Disorders, AKUADS = Aga Khan University Anxiety Depression Scale, PHQ = Patient Health Questionnaire, STAI = The State-Trait Anxiety Inventory, SCAN = Schedules for Clinical Assessment in Neuropsychiatry, CES-D = Centre for Epidemiological Studies Depression Scale

Outcome of Anxiety & Depression indicates that the measurement scale used does not discern between anxiety and depression and are used to measure both under as common mental health disorders

The review identified 12 social determinants: maternal age, maternal education, maternal occupation, household income, husband’s education, husband’s occupation, relationship with husband, intimate partner violence (IPV), pregnancy intentions, relationship with in-laws, social/family support and gender preference. Table 2 displays the overall trend of association for the social determinants.

**Table 2:** Summary of social determinants identified in the review.

Social determinant	Total number of studies	Number of studies included in univariate meta-analysis	Pooled OR (95% CI)	I^2^	Number of studies included in meta-analysis of AOR	Pooled AOR (95% CI)	I^2^
Maternal age	22	-	-	-	-	-	-
Maternal education/literacy	21	-	-	-	-	-	-
Maternal occupation	16	15	1.03 (0.76–1.41)	82%	2	1.33 (0.55–3.21)	73%
Household income	13	-	-	-	-	-	-
Husband’s education	7	-	-	-	-	-	-
Husband’s occupation	7	7	2.27 (0.96–5.41)	90%	2	0.85 (0.35–2.08)	83%
Relationship with husband	13	11	3.04 (1.63–5.68)*	95%	5	2.33 (0.90–6.02)	89%
Intimate Partner Violence	15	15	3.58 (2.57–5.00)*	61%	9	2.48 (1.41–4.33)*	71%
Unplanned pregnancy	20	19	1.88 (1.44–2.45)*	91%	5	1.53 (1.28–1.83)*	19%
Relationship with in-laws	7	7	4.23 (3.11–5.74)*	49%	3	2.69 (1.25–5.80)*	54%
Social/family support	10	9	2.14 (1.60–2.85)*	60%	4	1.42 (0.78–2.62)	75%
Male gender preference	6	6	1.84 (1.44–2.36)*	0%	3	3.06 (1.40–6.72)*	61%

*Statistically significant at 0.05 significance level

OR = Odds ratio

AOR = Adjusted Odds Ratio

### Maternal age

Twenty-two studies presented results on the age of the pregnant women (Table 3). The large variation in the reporting of maternal age made it unsuitable for meta-analysis. Six studies found women in the higher maternal age groups had significantly higher odds of antenatal depression [17, 30, 43] and co-morbid antenatal depression and anxiety [18, 26, 47] compared to women in the lower maternal age categories. Similarly, one study [31] found women with antenatal depression/anxiety had significantly higher mean maternal age compared to women without antenatal depression/anxiety. Conversely, three studies [32, 40, 44] found higher maternal age was significantly associated with lower comorbid antenatal depression & anxiety. Twelve studies [4, 15, 16, 35, 38, 46–48, 50–53] found no significant association. Nevertheless, despite non-significance, seven out of these twelve studies found an effect size that indicated higher maternal age groups had higher odds of antenatal depression/anxiety [4, 15, 16, 38, 46, 51, 53]. Whereas five studies found effect sizes that suggested the opposite trend [35, 47, 48, 50, 52]. Therefore, majority of the studies (n = 14) suggest a trend in higher risk with of antenatal depression/anxiety with higher maternal age.

**Table 3:** Association between antenatal depression/anxiety and maternal age in the included studies.

**Author & study year**	**Maternal age comparison groups**	**OR (95% CI)^a^**	**AOR (95% CI)^a^**
Ali et al 2012 [18]	<30 & ≥30	3.10 (1.29–7.42)*	3.54 (1.12–10.12)*
Ayyub et al 2018 [17]	15–20 & ≥32	3.03 (1.20–7.15)*	2.77 (1.13–6.82)*
Babu et al 2018 [47]	<25 & ≥25	1.34 (0.78–2.30)	0.73 (0.42–1.27)
Bavle et al 2016 [48]	<25 & ≥25	0.93 (0.41–2.08)	-
Dahiya et al 2020 [52]	<30 & ≥30	0.22 (0.03–1.68)	-
Gausia et al 2009 [53]	<35 & ≥35	1.19 (0.62–2.29)	-
Ghaffar et al 2017 [33]	<35 & ≥35	1.23 (1.13–1.62)*	-
Humayun et al 2013 [35]	<20 & ≥20	0.37 (0.05–3.00)	-
Jamal et al 2018 [38]	<25 & ≥25	1.43 (0.46–4.40)	-
Karmaliani et al 2009 [16]	<25 & ≥25	1.09 (0.83–1.44)	-
Nasreen et al 2011 [9]	<20 & ≥35	-	3.00 (1.12–8.01)*
Nath et al 2019 [15]	<20 & ≥20	1.25 (0.75–1.07)	-
Rahman et al 2003 [4]	<30 & ≥30	1.41 (0.94–2.11)	-
Safi et al 2013 [43]	<20 & ≥ 20	2.00 (1.10–3.62)*	-
Sheeba et al 2019 [46]	<20 & ≥20	1.16 (0.67–1.99)	-
Shidhaye et al 2017 [50]	<20 & ≥20	0.73 (0.39–1.35)	-
Srinivasan et al 2015 [51]	<20 & ≥20	1.39 (0.61–3.16)	-
Surkan et al 2018 [30]	<20 & ≥20	1.15 (1.03–1.30)*	1.38 (1.12–1.70)*
**Author & Study year**	**Mean maternal age (years) in depressed group**	**Mean maternal age (years) in non-depressed group**	**P-value**
Din et al 2016 [32]	23.30	24.44	0.038*
Mir et al 2012 [31]	26.30	24.30	0.003*
Niaz et al 2004 [40]	25.15	27.18	<0.05*
Shehroz et al 2019 [44]	25.15	27.18	<0.05*

^a^Lower age group is the reference group

OR = Odds Ratio

AOR = Adjusted Odds Ratio

*Statistically significant at 0.05 significance level

### Maternal education/literacy

Twenty-one studies presented results on maternal education and literacy (Table 4). Due to the variability in the reporting of education, meta-analysis was not appropriate. Nine studies found that women with the higher education/literacy levels had significantly lower odds of antenatal depression [9, 29–31, 39, 43, 45, 50, 52] compared to women with lower education/literacy levels. Conversely, two studies showed women with higher education/literacy levels had higher odds of antenatal depression [48] and co-morbid antenatal depression and anxiety [16]. Ten studies found no significant association [4, 15, 18, 33, 38, 44, 46, 47, 51, 53]. Out of these ten studies, nine studies [4, 15, 18, 33, 44, 46, 47, 51, 53] found a trend that indicated women with lower education/literacy levels had lower odds of antenatal depression/anxiety.

**Table 4:** Association between antenatal depression/anxiety and maternal education/literacy in the included studies.

**Author & study year**	**Maternal education/literacy level**	**OR (95% CI)**	**AOR (95% CI)**
Ali et al 2012 [18]	Graduate and above	REF	-
	Up to intermediate	0.87 (0.38–2.00)	
Ayaz et al 2019 [29]	College or university	REF	-
	Uneducated	2.12 (2.02–3.68)*	
Babu et al 2018 [47]	Attended college	REF	REF
	Did not attend college	0.70 (0.42–1.16)	0.81 (0.46–1.42)
Bavle et al 2016 [48]	Attended college	REF	-
	Did not attend college	0.46 (0.24–0.89)*	
Dahiya et al 2020 [52]	Illiterate	REF	-
	Literate	0.25 (0.11–0.58)*	
Gausia et al 2009 [53]	>5 years of education	REF	REF
	0–5 years of education	1.57 (1.01–2.44)*	0.99 (0.58–1.67)
Ghaffar et al 2017 [33]	No education	REF	-
	Primary	1.40 (0.70–2.12)	
	Secondary	1.03 (0.55–1.14)	
	Tertiary	1.19 (0.62–1.03)	
Jamal et al 2018 [38]	Intermediate/graduate and above	REF	-
	Primary and below	1.05 (0.47–2.37)	
Karmaliani et al 2009 [16]	None	REF	-
	1–9 years	1.52 (1.03–2.25)*	
	10 years or more	2.01 (1.30–3.10)*	
Maselko et al 2018 [39]	None	REF	-
	Primary	0.94 (0.62–1.42)	
	Secondary	0.44 (0.30–0.64)*	
	Tertiary	0.27 (0.17–0.44)*	
Mir et al 2012 [31]	Literate	REF	REF
	Illiterate	2.50 (1.56–4.03)*	1.83 (1.08–3.08)*
Nasreen et al 2011 [9]	Illiterate	-	REF
	Literate		0.59 (0.37–0.95)*
Nath et al 2019 [15]	Attended college	REF	-
	Did not attend college	0.86 (0.52–1.42)	
Rahman et al 2003 [4]	Literate	REF	-
	Illiterate	0.80 (0.60–1.00)	
Safi et al 2013 [43]	No education	REF	-
	Intermediate and above	0.18 (0.10–0.34)*	
Sheeba et al 2019 [46]	Attended college	REF	-
	Did not attend college	0.71 (0.40–1.25)	
Shidhaye et al 2017 [50]	Illiterate/completed primary	REF	-
	Junior college or above	0.30 (0.10–0.80)*	
Srinivasan et al 2015 [51]	≤10^th^ class	REF	-
	11^th^ -12^th^ class	0.78 (0.28–2.22)	
	Graduate and above	1.32 (0.51–3.46)	
Surkan et al 2018 [30]	≤10 years in education	REF	REF
	1–9 years	1.82 (1.37–2.40)*	1.45 (1.07–1.98)*
	None	2.87 (2.17–3.81)*	1.50 (1.05–2.15)*
Zia et al 2018 [45]	College or university	REF	-
	Uneducated	2.12 (2.0.2–3.63)*	
**Author & study year**	**Mean years in education in women with depression**	**Mean years in education in women without depression**	**P-value**
Shehroz et al 2019 [44]	6.9	6.5	>0.05

REF = reference group

OR = Odds Ratio

AOR = Adjusted odds ratio

*Statistically significant at 0.05 significance level

### Maternal occupation

**Fig 2:**
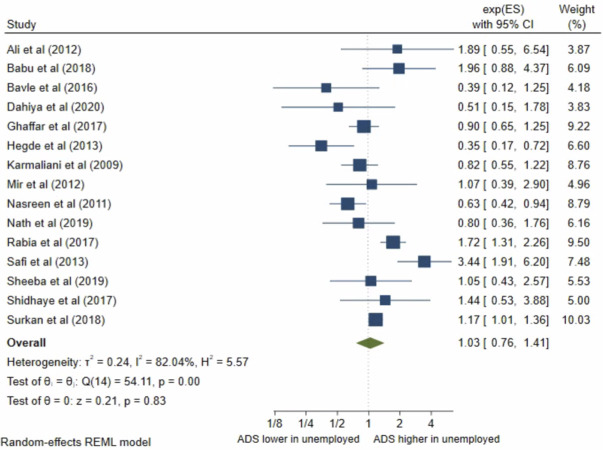
Forest plot of association between antenatal depression/anxiety and maternal occupation unadjusted OR.

**Fig 3:**
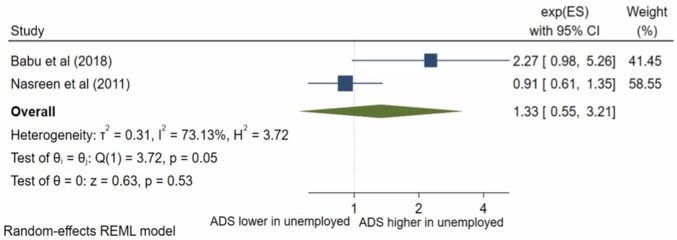
Forest plot of association between antenatal depression/anxiety and maternal occupation AOR.

Sixteen studies presented results on maternal occupation. Fifteen met random-effects meta-analysis criteria. The pooled OR (Fig 2) for antenatal depression/anxiety in unemployed women compared to employed was 1.03, (95% CI 0.76–1.41; I^2^ = 82%; n = 15 studies) and the pooled AOR (Fig 3) was 1.33, (95% CI 0.55–3.21; I^2^ = 73%, n = 2 studies). Both the pooled unadjusted and AORs indicate no significant association between antenatal depression/anxiety and maternal occupation. The one study, George et al [12], which was not eligible for inclusion in the meta-analysis also reported no significant association.

### Household income

Thirteen studies analysed household income (Table 5). Due to the variability in the income categories, meta-analysis was not appropriate. Three studies [33, 38, 43] found that women who live in households with lower levels of income had higher odds of antenatal depression compared to women living in households with higher levels of income. Similarly, a study [32] which analysed the mean monthly household income found that mean income was lower in those women who had co-morbid antenatal depression and anxiety compared to those women without. Nine studies found no significant association [4, 31, 36, 42, 47, 48, 50–52]. Of these nine studies, five studies [4, 47, 48, 51, 52] suggest a trend in reduced odds of antenatal depression/anxiety with lower household income levels, while four studies [31, 36, 42, 50] suggest the opposite trend.

**Table 5:** Association between antenatal depression/anxiety and household income in the included studies.

**Author & study year**	**Household income groups (rupees)**	**OR (95% CI)**	**AOR (95% CI)**
Babu et al 2018 [47]	>15,000	REF	REF
	10,001–15,000	0.91 (0.41–2.03)	0.88 (0.39–2.00)
	6001–10,000	1.18 (0.56–2.50)	1.02 (0.47–2.20)
	≤6K	0.52 (0.26–1.04)	0.49 (0.23–1.04)
Bavle et al 2016 [48]	>10,000	REF	-
	7501-10K	0.40 (0.17–0.94)*	
	5001–7500	0.21 (0.05–0.75)*	
	2501–5000	0.42 (0.13–1.36)	
	0–2500	0.62 (0.07–5.42)	
Dahiya et al 2020 [52]	≤13.51 USD	REF	-
	13.52–26.75 USD	39.00 (0.53–2883.6)	
	26.76–45.03 USD	13.00 (0.45–377.47)	
	45.04–90.07 USD	4.97 (0.21–109.19)	
	≥90.08 USD	3.56 (0.20–64.79)	
Ghaffar et al 2017 [33]	No income	REF	-
	<5000	0.33 (0.20–0.94)*	
	5000–15,0000	0.56 (0.34–0.88)*	
	>15,000	0.64 (0.17–0.99)*	
Imran et al 2010 [36]	>5000	REF	-
	<5000	1.99 (0.74–5.34)	
Jamal et al 2018 [38]	>25,000	REF	-
	≤25,000	6.24 (2.15–18.14)*	
Mir et al 2012 [31]	>10,000	REF	-
	5000–10,000	1.29 (0.68–1.45)	
	1,000–5,000	1.30 (0.72–2.36)	
	<1,000	1.57 (0.61–4.04)	
Rahman et al 2003 [4]	>2,500	REF	-
	<2,500	0.90 (0.60–1.30)	
Sabir et al 2019 [42]	≥50,000	REF	-
	25,000–50,000	1.44 (0.85–2.45)	
	<25,000	1.18 (0.69–2.02)	
Safi et al 2013 [43]	High	REF	-
	Middle	0.57 (0.26–1.25)	
	Low	2.83 (1.25–6.41)*	
Shidhaye et al 2017 [50]	Low	REF	-
	Middle	0.40 (0.10–1.00)	
	High	0.60 (0.30–1.20)	
Srinivasan et al 2015 [51]	>1 Lakh	REF	-
	50,000–1 lakh	0.49 (0.19–1.22)	
	≤50,000	0.70 (0.15–3.25)	
**Author & study year**	**Mean monthly household income (thousand rupees) in depressed group**	**Mean monthly household income (rupees) in non-depressed group**	**P-value**
Din et al 2016 [32]	24.91	27.41	0.041*

REF = Reference group

OR = Odds Ratio

AOR = Adjusted Odds Ratio

*Statistically significant at 0.05 significance level

### Husband’s education/literacy

Seven studies presented results on husband’s education/literacy (Table 6). Due to the variability in the categories for education, meta-analysis was deemed inappropriate. Two studies [4, 39] found that women whose husbands were illiterate had higher odds of antenatal depression compared to women whose husbands were literate (Table 5). Five studies found no significant association [15, 29, 45–47], however, they all suggest a trend in higher odds of antenatal depression in women with husband’s who have lower levels of education.

**Table 6:** Association between antenatal depression/anxiety and husband’s education/literacy in the included studies.

Author & study year	Husband’s education/literacy level	OR (95% CI)	AOR (95% CI)
Ayaz et al 2019 [29]	College or university	REF	-
	Uneducated	1.24 (0.30–5.24)	
Babu et al 2018 [47]	Attended college	REF	-
	Did not attend college	1.22 (0.75–1.98)	
Maselko et al 2018 [39]	None	REF	-
	Primary	0.89 (0.50–1.59)	
	Secondary	0.50 (0.31–0.78)*	
	Tertiary	0.25 (0.13–0.49)*	
Nath et al 2019 [15]	Attended college	REF	-
	Did not attend college	1.22 (0.71–2.11)	
Rahman et al 2003 [4]	Literate	REF	-
	Illiterate	1.60 (1.20–2.20)*	
Sheeba et al 2019 [46]	Attended college	REF	-
	Did not attend college	1.26 (0.77–2.05)	
Zia et al 2018 [45]	College or university	REF	-
	Uneducated	1.24 (0.30–1.34)	

REF = reference group

OR = Odds Ratio

AOR = Adjusted odds ratio

*Statistically significant at 0.05 significance level

### Husband’s occupation

**Fig 4:**
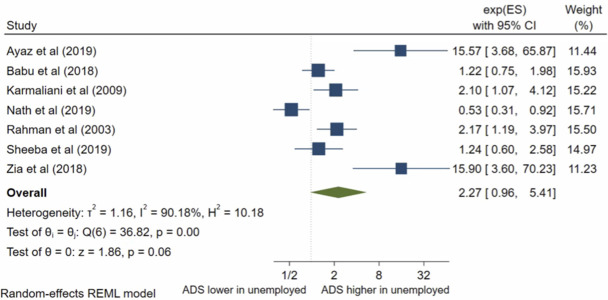
Forest plot of association between antenatal depression/anxiety and husband’s occupation unadjusted OR.

**Fig 5:**
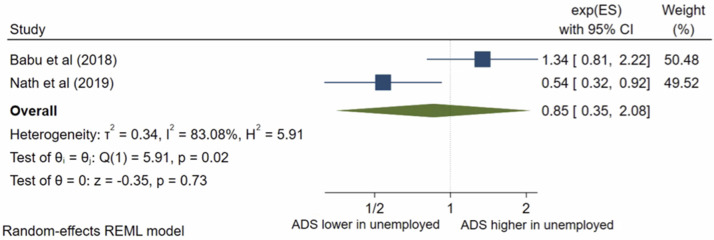
Forest plot of association between antenatal depression/anxiety and husband’s occupation AOR.

Seven studies analysed husband’s occupation; all were eligible for inclusion into the meta-analysis. The pooled OR (Fig 4) for antenatal depression/anxiety in women whose husbands were unemployed compared to women whose husbands were employed was 2.27, (95% CI 0.96–5.41; I^2^ = 90%; n = 7 studies). The pooled AOR (Fig 5) was 0.85, (95% CI 0.35–2.08; I^2^ = 83%; n = 2 studies). There was evidence of publication bias (p = 0.0001) in the adjusted analysis. Both the pooled unadjusted OR and AOR suggest no significant association between antenatal depression/anxiety and husband’s occupation.

### Relationship with husband

**Fig 6:**
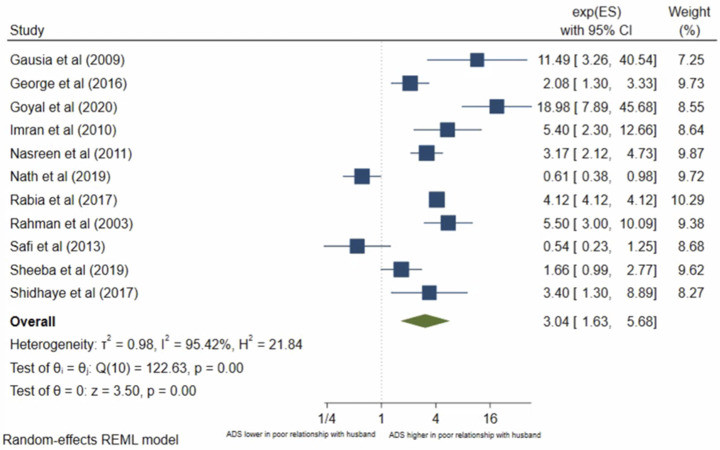
Forest plot of association between antenatal depression/anxiety and relationship with husband unadjusted OR.

**Fig 7:**
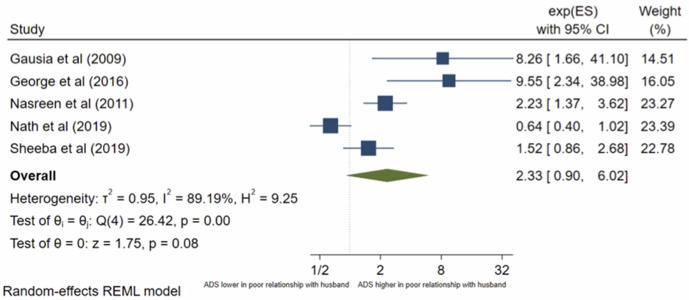
Forest plot of association between antenatal depression/anxiety and relationship with husband AOR.

Thirteen studies analysed a women’s relationship with her husband. Relationship with husband was mostly reported as a binary variable: poor vs good relationship, but two studies, Mir et al [31] and Srinivasan et al [51] reported it as a continuous variable using a martial satisfaction scale. Both found that the mean marital satisfaction score was significantly lower in women with antenatal depression compared to women without antenatal depression. The other 11 studies were eligible for inclusion in the meta-analysis. The pooled OR (Fig 6) for antenatal depression/anxiety in women who report a poor relationship with their husband compared to women who report a good relationship was 3.04, (95% CI 1.63–5.68; I^2^ = 95%, n = 11 studies). The pooled AOR (Fig 7) was 2.33, (95% CI 0.90–6.02; I^2^ = 89%, n = 5 studies). Publication bias was evident from the Eggers test (p = 0.0075) for both analyses. The pooled unadjusted OR suggests women who report a poor relationship have significantly higher odds of antenatal depression/anxiety compared to women who report a good relationship, however, this was no longer significant in the adjusted analysis. To assess the different significance level of the pooled OR and pooled AOR, sub-group analysis was conducted to compare the pooled raw OR and pooled AOR including only the five studies that reported both a raw OR and AOR. The sub-group analysis found that when pooling only these studies, the pooled OR was 1.82 (95% CI 1.45–2.28, I^2^ = 89%, n = 5), which is still significant. This suggests that the non-significant pooled AOR is not due to the different number of studies, the study characteristics or sample size, but likely due to the confounding factors.

### Intimate partner violence

**Fig 8:**
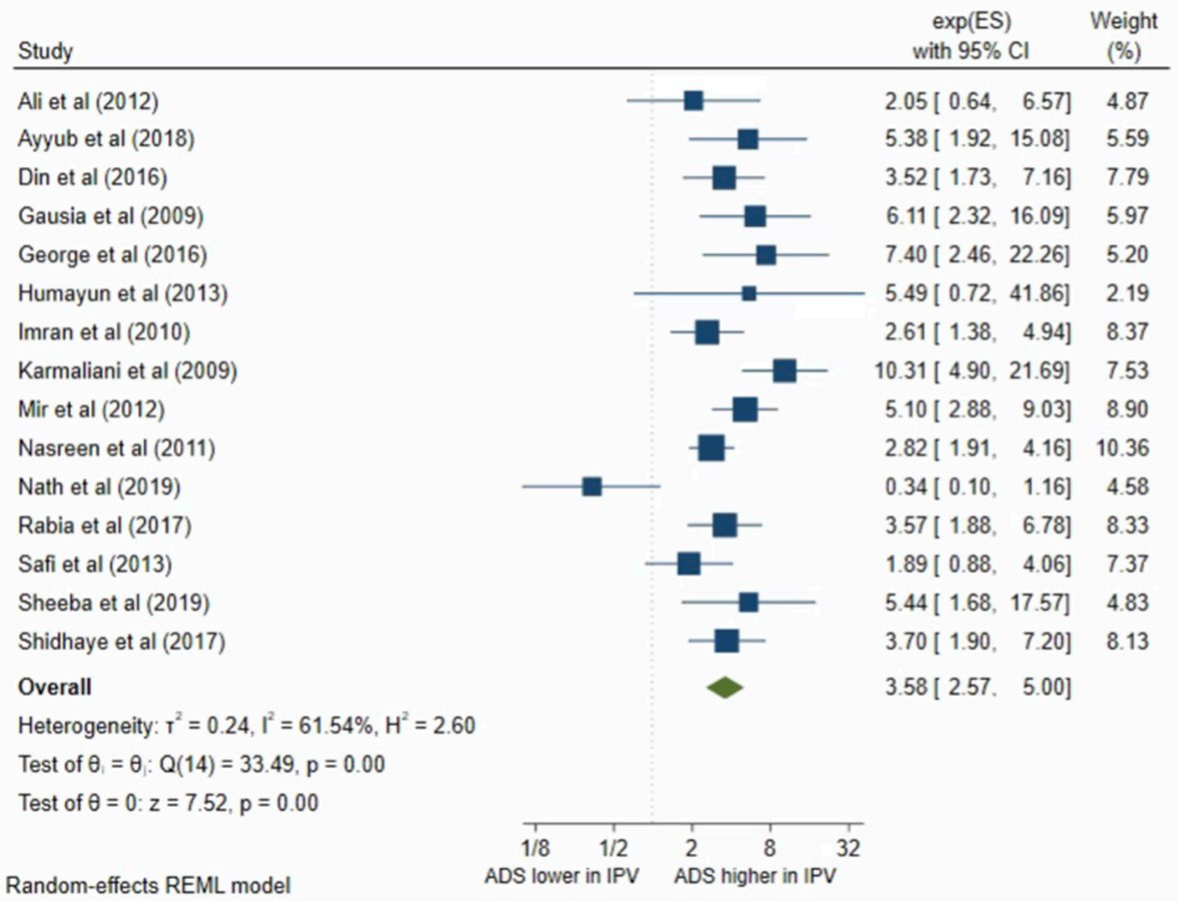
Forest plot of association between antenatal depression/anxiety and IPV unadjusted OR.

**Fig 9:**
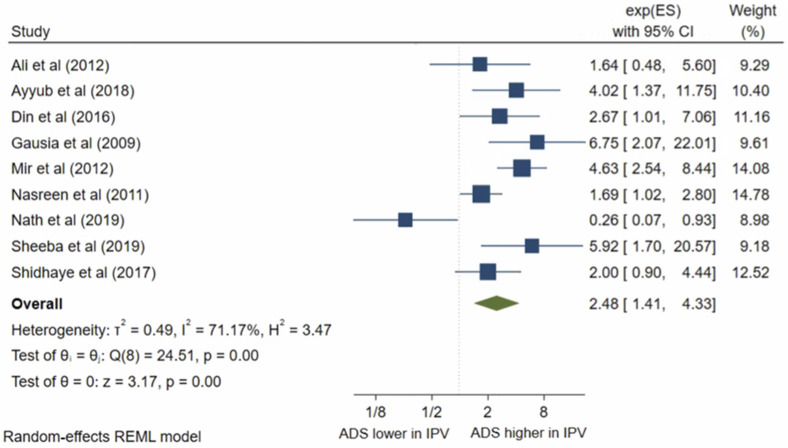
Forest plot of association between antenatal depression/anxiety and IPV AOR.

Fifteen studies presented results on IPV during pregnancy. All studies met the criteria for inclusion in the meta-analysis. The pooled OR (Fig 8) for antenatal depression in women who had suffered from IPV during pregnancy compared to those who had not was 3.58, (95% CI 2.57–5.00; I^2^ = 61%; n = 15 studies). The pooled AOR (Fig 9) was 2.48, (95% CI 1.41–4.33; I^2^ = 71%; n = 9 studies). Both the unadjusted OR and AOR suggest that women who experienced IPV during pregnancy have significantly higher odds of antenatal depression/anxiety compared to women who have not experienced IPV during their pregnancy.

### Unplanned pregnancy

**Fig 10:**
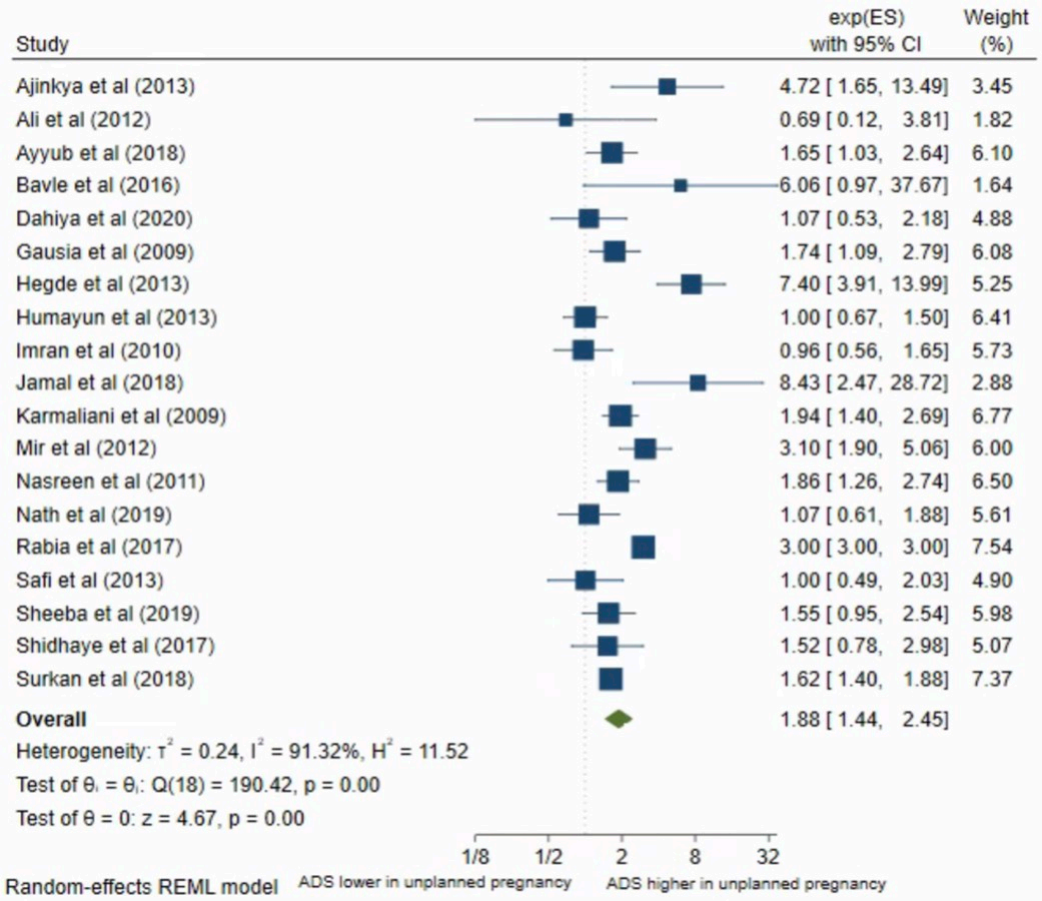
Forest plot of association between antenatal depression/anxiety and pregnancy intentions unadjusted OR.

**Fig 11:**
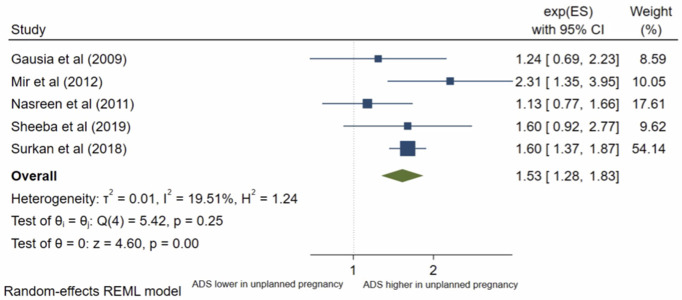
Forest plot of association between antenatal depression/anxiety and pregnancy intentions AOR.

Twenty studies analysed pregnancy intentions. George et al [12] was not eligible for inclusion into meta-analysis; this study found no significant association. Nineteen studies were included in the meta-analysis. The pooled OR (Fig 10) for antenatal depression/anxiety in women who had an unplanned pregnancy compared to women with planned pregnancy was 1.88, (95% CI 1.44–2.45; I^2^ = 91%; n = 19 studies). The pooled AOR (Fig 11) was 1.53, (95% CI 1.28–1.83; I^2^ = 19%; n = 5 studies). Both the pooled unadjusted and AOR suggest that women who had unplanned pregnancies have higher odds of antenatal depression/anxiety compared to women who had planned pregnancies.

### Relationship with in-laws

**Fig 12:**
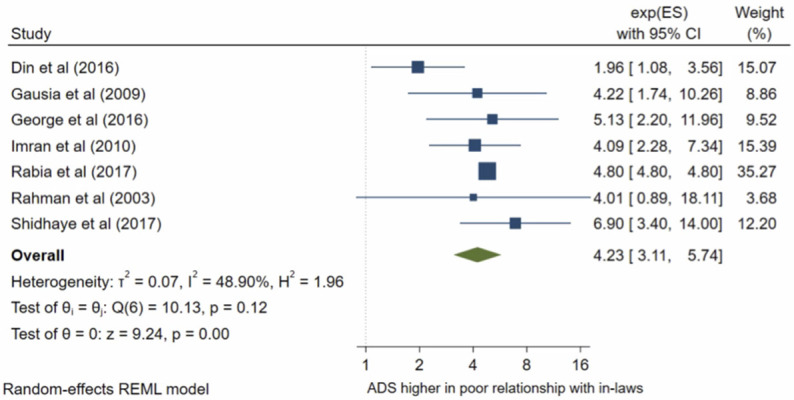
Forest plot of association between antenatal depression/anxiety and relationship with in-laws unadjusted OR.

**Fig 13:**
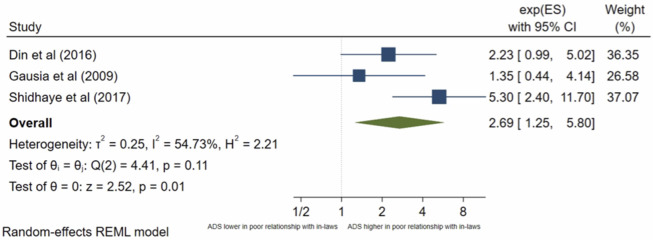
Forest plot of association between antenatal depression/anxiety and relationship with in-laws AOR.

Seven studies analysed a women’s relationship with her in-laws. The pooled OR (Fig 12) for antenatal depression in women with a poor relationship with in-laws compared with those with a good relationship was 4.23, (95% CI 3.11–5.74; I^2^ = 49%; n = 7 studies). The pooled AOR (Fig 13) was 2.69, (95% CI 1.25–5.80; I^2^ = 54%; n = 3 studies). Both the unadjusted and AOR suggest that a poor relationship with in-laws during pregnancy is significantly associated with higher odds of antenatal depression compared to perceiving a good relationship with in-laws.

### Social/family support

**Fig 14:**
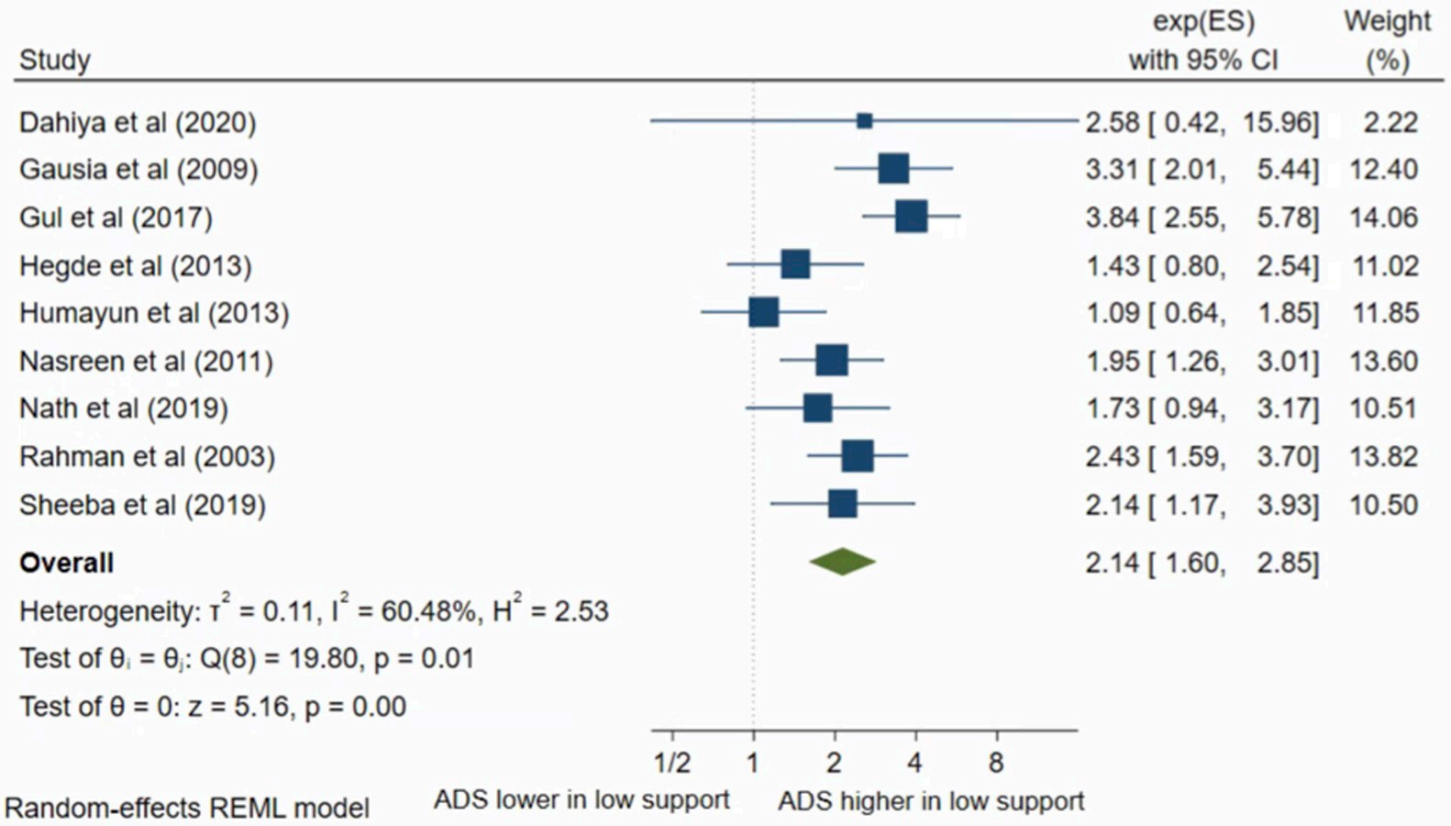
Forest plot of association between antenatal depression/anxiety and social/family support unadjusted OR.

**Fig 15:**
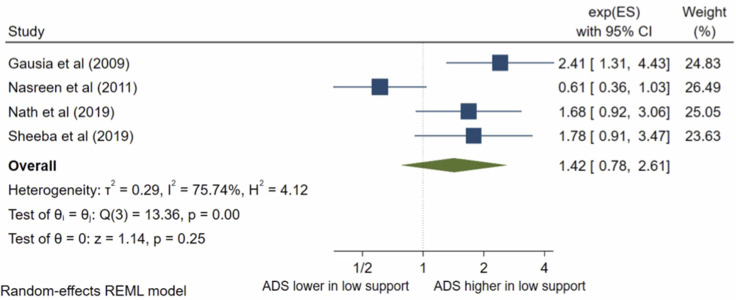
Forest plot of association between antenatal depression/anxiety and social/family support AOR.

There were ten studies which analysed social support. Nine studies were eligible for inclusion in the meta-analysis. The pooled OR (Fig 14) for antenatal depression/anxiety in women with low social/family support compared to women with high social/family support was 2.14, (95% CI 1.60–2.85; I^2^ = 60%; n = 9 studies). The pooled AOR (Fig 15) was 1.42, (95% CI 0.78–2.62; I^2^ = 75%; n = 4). The pooled unadjusted OR suggests that women with low social and family support had higher odds of antenatal depression/anxiety compared to women with high support, however, this was no longer significant in the adjusted analysis. To assess the different significance level of the pooled OR and pooled AOR, sub-group analysis was conducted to compare the pooled raw OR and pooled AOR including only the four studies that reported both a raw OR and AOR. The sub-group analysis found that when pooling only these studies, the pooled OR was 2.24 (95% CI 1.73–2.91, I^2^ = 75%, n = 4), which is still significant. This suggests that the non-significant pooled AOR is not due to the different number of studies, the study characteristics or sample size, but likely due to the confounding factors.

### Gender preference

**Fig 16:**
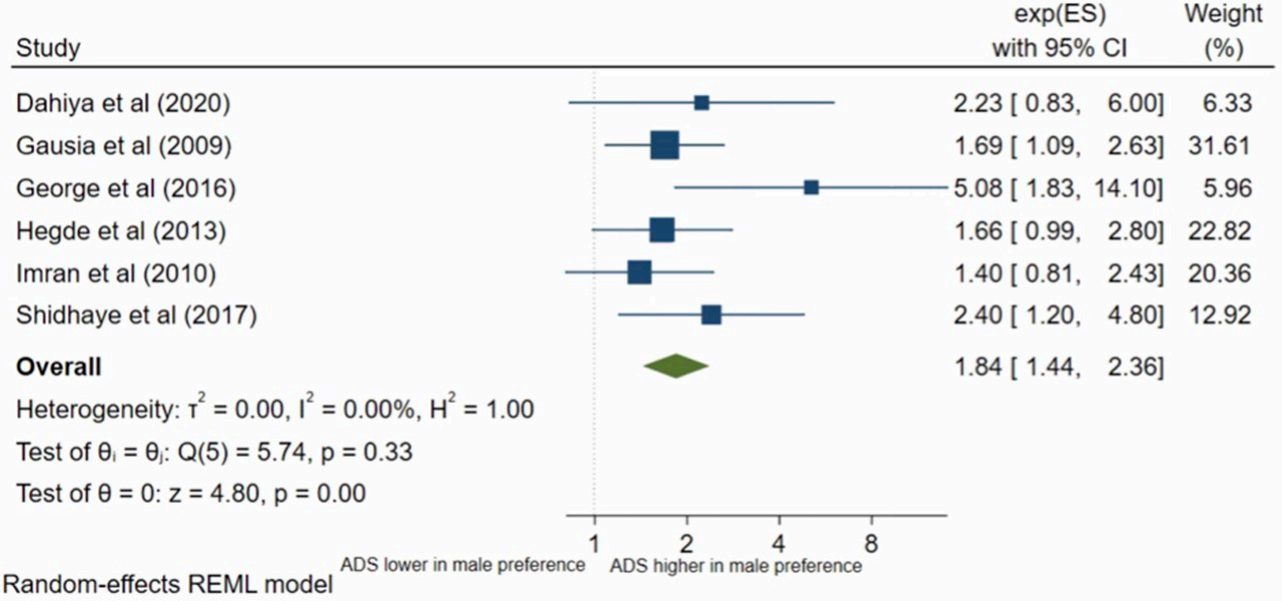
Forest plot of association between antenatal depression/anxiety and gender preference unadjusted OR.

**Fig 17:**
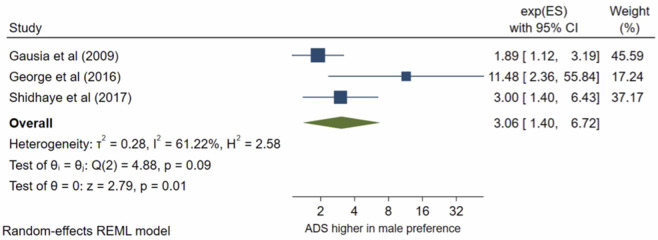
Forest plot of association between antenatal depression/anxiety and gender preference AOR.

Six studies analysed gender preference for the baby. All the studies met the inclusion criteria for meta-analysis. The pooled OR (Fig 16) of antenatal depression in women who wanted a male child compared to wanting a female child was 1.84, (95% CI 1.44–2.36; I^2^ = 0%, n = 6 studies). The pooled AOR (Fig 17) was 3.06, (95% CI 1.40–6.72; I^2^ = 61%, n = 3 studies). Both the pooled unadjusted OR and AOR indicate that a male gender preference was significantly associated with increased odds of antenatal depression compared to wanting a female child.

### Sensitivity analysis

Analyses were repeated by removing the studies which scored low (0–3) in the quality assessment. The findings only significantly changed for husband’s occupation. When removing the low-quality study, Zia et al [45], from the unadjusted analysis, the results were no longer significant (OR 1.65, 95% CI 0.88–3.10). Nevertheless, Zia et al [45] did not carry out adjusted analysis, therefore, the adjusted analysis in the meta-analysis for husband’s occupation remains the same, suggesting no significant association between husband’s occupation and antenatal depression.

## Discussion

To our knowledge this is the first systematic review and meta-analysis to investigate the social determinants of antenatal depression and anxiety in Bangladesh, India and Pakistan. The meta-analysis found IPV, preference for a male child, poor relationship with in-laws and unplanned pregnancy to be statistically significantly associated with antenatal depression and anxiety.

Previous reviews [6, 19, 20] have consistently found IPV to be an important risk factor for antenatal depression/anxiety globally, supporting the finding in this present review. This may be linked to the social, emotional and physical isolation and pressure pregnant women experience when suffering from IPV [6]. Particularly in LMICs, where IPV rates are higher compared to HICs [6], women are subject to social and cultural norms which prevent them from sharing such information [54]. Furthermore, there may be forced economic dependency, which is associated with IPV, on the perpetrator where many women in LMICs are not able to leave their partners [55]. One study found that many women in South Asian communities do not consider their relationship with their husband to be poor even if the woman faces challenges such as IPV [9]. There are many underlying mechanisms within the relationship with husband which should be further investigated through qualitative studies.

The present review found unplanned pregnancy to be a significant social determinant of antenatal depression and anxiety which is reflected in previous reviews [19, 20]. Unplanned pregnancies can reflect a lack of access to adequate family planning services [56] and power relations between the husband and wife leading to IPV through forced sex without the use of contraception [57]. These issues can cause significant distress to pregnant women. Further, Lee et al [58] and Marchesi et al [59], suggest that unwanted pregnancies, which is a pregnancy intention subset of unplanned pregnancy [60], were a significant predictor of antenatal depression, but mostly in the first trimester, with less importance over time. This may reflect the initial stress of an unwanted pregnancy reducing throughout the pregnancy as the woman comes to terms with the idea of having a baby. This is an interesting finding which has not been investigated in any of the included studies in this review suggesting a need for further research on this within these South Asian countries.

Many studies focus on the relationship with husband, while neglecting the crucial interactions between in-laws and the expecting mother. Particularly in South Asian countries, where women are often expected to live with their in-laws after marriage and throughout [61], these vital family dynamics can shape how women experience pregnancy. Although the pooled OR and AOR showed a significant association between poor relationship with in-laws and antenatal depression/anxiety, only three studies [32, 50, 53] went onto carry out multivariate analysis of which two [32, 53] found a non-significant association. The study with the largest weight, Shidhaye et al [50], which largely contributed to the significant association, was assessed to be a high-quality study. However, differences in the factors adjusted for in the multivariate analysis may contribute to the differences in the results between the three studies.

Gender preference for the child appears to be a relatively under-researched determinant in relation to antenatal depression/anxiety, as highlighted by the small number of studies included in this review which addressed it, despite it being a common societal issue within these South Asian countries [62]. The significant association between preferring a male child and antenatal depression/anxiety found in this review is reflected in observational studies conducted in China [63] and Iran [64]. Male children are considered a blessing as they carry the family name and can provide for their parents in old age, whereas female children are viewed as a burden for the family [61]. Hence, indirect and direct pressure, along with internalised expectations, from the family and society to have a male child may produce adverse effects on the mental health of pregnant women.

### Strengths and limitations of the study

A wide range of databases were used to systematically search for papers between January 2000 and January 2021 using the PICOS framework. The date restriction was imposed because an initial search of Medline showed no appropriate studies before 2000, suggesting that investigation of risk factors of maternal depression in South Asia is a relatively recent area of research interest. Overtime clinical approaches and thinking towards maternal depression and depression as a whole, particularly in LMICs, have developed and changed [65]. Therefore, a date restriction ensured that the literature in this review would still be clinically and socially relevant. Supplementary searches such as hand searching reference lists, citation searching and contacting authors, were carried out to provide a comprehensive review on a large number of social determinants. Procedures to minimise human error and subjectivity included double independent screening, data extraction and quality assessment. The included studies were mostly of high or medium quality suggesting lower levels of bias within the studies. Only seven out of the 33 studies were assessed as low quality and after these were removed in the sensitivity analysis to determine their influence, no significant changes were identified within the results. Although there are differences between the different depression and anxiety scales, this review aimed to further limit bias by only including studies which used validated measurement scales to determine depression and anxiety and questionnaires which were standardised. This allowed for more appropriate comparisons to be made between the included studies.

Publication bias was found in the meta-analyses for husband’s occupation and relationship with husband, therefore, the results should be interpreted with caution. Nevertheless, the results from the meta-analyses for these two factors were non-significant which suggests that the results from the included studies are not biased towards a particular direction, given the non-significant pooled AORs.

Heterogeneity was found to be high when a large number of studies were pooled. This could be due to methodological differences such as sampling of participants, sample size, use of different measurement tools and difference in characteristics between Bangladesh, India and Pakistan. Although these three countries share similar social and cultural norms and characteristics which deems it appropriate to pool them together, there is heterogeneity between them. There are differences in the predominant religions found in the three countries. In India, approximately 80% of the population are Hindu, followed by Muslims who make up 13% of the population [66]. Conversely, Bangladesh is made up 81% Muslims and 10% Hindus. There are certain differences between these religions which can influence some of the social determinants investigated in this review. For example, the system of dowry (money or assets given to the groom’s family by the bride’s family) is more common in Hinduism and is a prominent reason why many families prefer having male children. Therefore, male gender preference may be more prevalent in India than in Bangladesh adding to the heterogeneity. This review used random-effects model to account for the heterogeneity.

Finally, this review restricted the included studies to those published in or containing English translations since producing translations was not feasible. Therefore, studies published in languages native to Bangladesh, India and Pakistan may have been missed from this review. Nevertheless, journals from Bangladesh, India and Pakistan tend to increasingly publish in the English language or contain translations making them accessible for a wider audience.

## Conclusion

This systematic review and meta-analysis found that IPV, male gender preference, poor relationship with in-laws and unplanned pregnancies were significant predictors of antenatal depression in Bangladesh, India and Pakistan. The findings are important for the development of targeted interventions for the prevention and screening of antenatal depression and anxiety in South Asia. These social determinants highlight the inequalities between gender where many women are victims of IPV, forced sex and unplanned pregnancies, and male children are often preferred over female children. Such inequalities should be tackled through policy, education and advocacy within South Asian communities. Further research involving large scale studies incorporating factors which this review found inconsistent evidence on and qualitative studies to understand the relationships are needed.

## Supporting information

S1 Checklist PRISMA 2009 checklist. (DOC)

S1 Fig Search terms for OVID database. (DOCX)

S2 Fig Adapted Newcastle-Ottawa Scale for cross-sectional studies. (DOCX)

S1 Table MOOSE checklist for meta-analysis of observational studies. (DOCX)

S2 Table Data extraction protocol. (DOCX)

S3 Table Quality scores for all included studies. (DOCX)

S4 Table Results for social determinants investigated by only one study. (DOCX)

S5 Table Factors included in the included studies’ adjusted models. (DOCX)
