# Supplementary material for: Social determinants of antenatal depression and anxiety among women in South Asia: A systematic review & meta-analysis
Source: PLoS One. 2022 Feb 9;17(2):e0263760. doi: 10.1371/journal.pone.0263760 (PMC8827460; doi:10.1371/journal.pone.0263760)
Supplement: S1 Table — (DOCX) [file pone.0263760.s004.docx]

**S1 Table. MOOSE checklist (1) for Meta-analysis of Observational Studies**

| **Item No** | **Recommendation** | **Reported yes/no** | **Reported in section, page number** |
| --- | --- | --- | --- |
|  | **Reporting of background should include** | | |
| 1 | Problem definition | Yes | Introduction, 3 |
| 2 | Hypothesis statement | No | N/A |
| 3 | Description of study outcome(s) | Yes | Methods, 4-5 |
| 4 | Type of exposure or intervention used | Yes | Methods, 4-5 |
| 5 | Type of study designs used | Yes | Methods, 4 |
| 6 | Study population | Yes | Methods, 4 |
|  | **Reporting of search strategy should include** | | |
| 7 | Qualifications of searchers (eg, librarians and investigators) | Yes | Methods, 4 |
| 8 | Search strategy, including time period included in the synthesis and key words | Yes | Methods, 4 &  S1 Fig |
| 9 | Effort to include all available studies, including contact with authors | Yes | Methods, 5 |
| 10 | Databases and registries searched | Yes | Methods, 4-5 |
| 11 | Search software used, name and version, including special features used (eg, explosion) | Yes | Methods, 4-5  S1 Fig |
| 12 | Use of hand searching (eg, reference lists of obtained articles) | Yes | Methods, 5 |
| 13 | List of citations located and those excluded, including justification | Yes | Results, 6  Fig 1 |
| 14 | Method of addressing articles published in languages other than English | Yes | Methods, 4  Discussion, 31 |
| 15 | Method of handling abstracts and unpublished studies | Yes | Methods, 4-5 |
| 16 | Description of any contact with authors | No | N/A |
|  | **Reporting of methods should include** | | |
| 17 | Description of relevance or appropriateness of studies assembled for assessing the hypothesis to be tested | No | N/A |
| 18 | Rationale for the selection and coding of data (eg, sound clinical principles or convenience) | Yes | Methods, 5-6 |
| 19 | Documentation of how data were classified and coded (eg, multiple raters, blinding and interrater reliability) | Yes | Methods, 5-6 |
| 20 | Assessment of confounding (eg, comparability of cases and controls in studies where appropriate) | Yes | Methods, 5-6 |
| 21 | Assessment of study quality, including blinding of quality assessors, stratification or regression on possible predictors of study results | Yes | Methods, 5  S2 Table, S2 Fig |
| 22 | Assessment of heterogeneity | Yes | Methods, 5  Results |
| 23 | Description of statistical methods (eg, complete description of fixed or random effects models, justification of whether the chosen models account for predictors of study results, dose-response models, or cumulative meta-analysis) in sufficient detail to be replicated | Yes | Methods, 5-6 |
| 24 | Provision of appropriate tables and graphics | Yes | Table 1  Fig 1  Supplementary info |
|  | **Reporting of results should include** | | |
| 25 | Graphic summarizing individual study estimates and overall estimate | Yes | Fig 2-17 |
| 26 | Table giving descriptive information for each study included | Yes | Table 1 |
| 27 | Results of sensitivity testing (eg, subgroup analysis) | Yes | Results, 28-29 |
| 28 | Indication of statistical uncertainty of findings | Yes | Confidence intervals provided throughout narrative results, tables and figures in the main manuscript and in Supplementary info |
|  | **Reporting of discussion should include** | | |
| 29 | Quantitative assessment of bias (eg, publication bias) | Yes | Results  Discussion, 31 |
| 30 | Justification for exclusion (eg, exclusion of non-English language citations) | Yes | Discussion, 31 |
| 31 | Assessment of quality of included studies | Yes | Results, 6  S3 Table |
|  | **Reporting of conclusions should include** | | |
| 32 | Consideration of alternative explanations for observed results | Yes | Conclusion, 32 |
| 33 | Generalization of the conclusions (ie, appropriate for the data presented and within the domain of the literature review) | Yes | Discussion, 29-30 |
| 34 | Guidelines for future research | Yes | Discussion, 29-32 |
| 35 | Disclosure of funding source | Yes | Funding declaration |

**References:**

1. Stroup DF, Berlin JA, Morton SC, et al, for the Meta-analysis Of Observational Studies in Epidemiology (MOOSE) Group. Meta-analysis of Observational Studies in Epidemiology. A Proposal for Reporting. *JAMA*. 2000;283(15):2008-2012.
