# Supplementary material for: Social determinants of antenatal depression and anxiety among women in South Asia: A systematic review & meta-analysis
Source: PLoS One. 2022 Feb 9;17(2):e0263760. doi: 10.1371/journal.pone.0263760 (PMC8827460; doi:10.1371/journal.pone.0263760)
Supplement: S2 Table — (DOCX) [file pone.0263760.s005.docx]

**S2 Table. Data extraction protocol**

Adapted from Cochrane data extraction template

| **Reviewer** |  |
| --- | --- |
| **Title of paper** |  |
| **Author and year** |  |
| **Country** | Bangladesh  India  Pakistan |
| **Methodology** | Cohort  Case-control  Cross-sectional |
| **Setting** | Hospital  Community-based |
| **Inclusion criteria for women** |  |
| **Exclusion criteria for women** |  |
| **Recruitment and sampling method** |  |
| **Sample size of pregnant women** |  |

| **Outcome** | **Ascertainment of outcome (what tool was used)** | **Was outcome tool validated?** | **What cut-off score was used to categorise the outcome** |
| --- | --- | --- | --- |
| Depression  Anxiety  Depression & Anxiety |  |  |  |

| **Social determinants collected from participants** | **Ascertainment of social determinants (e.g questionnaires etc)** | **Which social determinants included in analysis?** |
| --- | --- | --- |
|  |  |  |

**Results**

| **Social determinant** | **Unadjusted (*=significant)** | **Adjusted (*=significant)** | **Factors adjusted for** |
| --- | --- | --- | --- |
|  |  |  |  |
