# Supplementary material for: Social determinants of antenatal depression and anxiety among women in South Asia: A systematic review & meta-analysis
Source: PLoS One. 2022 Feb 9;17(2):e0263760. doi: 10.1371/journal.pone.0263760 (PMC8827460; doi:10.1371/journal.pone.0263760)
Supplement: S3 Table — (DOCX) [file pone.0263760.s006.docx]

**S3 Table. Quality scores for all included studies**

| **Paper** | **Newcastle Ottawa Scale question number and score allocated^a^** | | | | | | | |  | **Independent reviewer initials^d^** |
| --- | --- | --- | --- | --- | --- | --- | --- | --- | --- | --- |
|  | **1** | **2** | **3** | **4** | **5** | **6** | **7** | **Total stars^b^** | **Category^c^** |  |
| **Ajinkya 2013** | a* | a* | c | c | c | a** | b | 4 | Medium | NI & AW |
| **Ali 2012** | d | b | c | b* | a** | a** | a* | 6 | Medium | NI & AW |
| **Ayaz 2019** | d | b | c | b* | c | c* | a* | 3 | Low | NI &AW |
| **Ayyub 2018** | b* | a* | a* | a** | a** | a** | a* | 10 | High | NI & AW |
| **Babu 2018** | a* | a* | c | a** | a** | c* | a* | 8 | High | NI & AW |
| **Bavle 2016** | d | b | c | a** | c | a** | b | 4 | Medium | NI & AW |
| **Dahiya 2020** | a* | b | c | b* | b* | a** | a* | 6 | Medium | NI & AW |
| **Din 2016** | d | b | c | b* | a** | c* | a* | 5 | Medium | NI & AW |
| **Gausia 2009** | a* | a* | c | b* | a** | a** | a* | 8 | High | NI & AW |
| **George 2016** | a* | a* | a* | a** | a** | a** | a* | 10 | High | NI & AW |
| **Ghaffar 2017** | a* | a* | b | b* | a** | a** | a* | 8 | High | NI & AW |
| **Goyal 2020** | b* | a* | a* | b* | c | a** | b | 6 | Medium | NI & SF |
| **Gul 2017** | b* | a* | a* | a** | c | a** | b | 7 | Medium | NI & SF |
| **Hedge 2013** | d | b | c | b* | c | c* | b | 2 | Low | NI & SF |
| **Humayun 2013** | a* | a* | c | b* | c | c* | a* | 5 | Medium | NI & AW |
| **Imran 2010** | b* | a* | a* | b* | c | a** | b | 6 | Medium | NI & SF |
| **Jafri 2017** | a* | b | c | c | c | c* | b | 2 | Low | NI & SF |
| **Jamal 2018** | a* | a* | c | b* | b* | c* | b | 5 | Medium | NI & SF |
| **Karmaliani 2009** | d | b | c | b* | a** | a** | a* | 6 | Medium | NI & AW |
| **Maselko 2018** | a* | b | c | a** | b* | a** | a* | 7 | Medium | NI & SF |
| **Mir 2012** | a* | a* | a* | a** | a** | c* | a* | 9 | High | NI & SF |
| **Nasreen 2011** | a* | a* | a* | a** | a** | a** | a* | 10 | High | NI & AW |
| **Nath 2019** | a* | b | c | a** | a** | a** | a* | 8 | High | NI & AW |
| **Niaz 2004** | a* | b | c | c | c | c* | b | 2 | Low | NI & AW |
| **Rabia 2017** | b* | a* | a* | b* | c | c* | b | 5 | Medium | NI & AW |
| **Rahman 2003** | a* | b | c | a** | a** | c* | a* | 7 | Medium | NI & AW |
| **Sabir 2019** | b* | a* | a* | c | c | c* | b | 4 | Medium | NI & AW |
| **Safi 2013** | d | b | c | b* | c | a** | b | 3 | Low | NI & AW |
| **Sheeba 2019** | a* | b | c | a** | a** | a** | a* | 8 | High | NI & AW |
| **Shehroz 2019** | d | b | c | c | c | c* | b | 1 | Low | NI & AW |
| **Shidhaye 2017** | a* | a* | a* | a** | a** | a** | a* | 10 | High | NI & AW |
| **Srinivasan 2015** | b* | b | c | a** | c | a** | a* | 6 | Medium | NI & AW |
| **Surkan 2018** | a* | a* | a* | b* | a** | a** | a* | 9 | High | NI & AW |
| **Zia 2018** | d | b | c | b* | c | c* | b | 2 | Low | NI & AW |

^a^Newcastle-Ottawa question numbers 1-7, answers a-d, and associated number of stars (*) are detailed in Appendix

^b^Minimum number of possible stars to be awarded = 0, maximum number of possible stars to be awarded = 10.

^c^Categories were allocated as: Low = 0-3 stars, Medium = 4-7 stars, High = 8-10 stars.

^d^Reviewers initials relate to manuscript authors: NI, Nafisa Insan; AW, Anthony Weke, SF, Simon Forrest
