# Supplementary material for: Social determinants of antenatal depression and anxiety among women in South Asia: A systematic review & meta-analysis
Source: PLoS One. 2022 Feb 9;17(2):e0263760. doi: 10.1371/journal.pone.0263760 (PMC8827460; doi:10.1371/journal.pone.0263760)
Supplement: S4 Table — (DOCX) [file pone.0263760.s007.docx]

**S4 Table** Results for social determinants investigated by only one study

| **Social determinant** | **Groups** | **OR** | **AOR** |
| --- | --- | --- | --- |
| Number of people living in the same household | Up to 5  More than 5 | REF  1.38 (0.63-3.02) | - |
| Household decision maker | Self or husband  In-laws  Combined | REF  2.26 (0.75-6.83)  0.97 (0.41-2.30) | REF  3.81 (1.13-12.83)*  1.36 (0.51-3.59) |
| Death of some close relative | No  Yes | REF  1.94 (1.24-3.14)* | REF  1.95 (1.20-3.19)* |
| Food insecurity | No  Yes | REF  2.96 (1.92–4.57)* | REF  2.58 (1.64–4.08)* |
| Family type | Nuclear  Joint | REF  2.08 (1.17-3.69)* | REF  2.14 (1.00-4.57)* |
| Non-arranged marriage | No  Yes | REF  3.06 (1.19–7.87)* | REF  6.05 (1.72–21.23)* |
| Fear of childbirth | No  Yes | REF  1.52 (1.00-2.31)* | - |
| Separation from husband | No  Yes | REF  2.28 (1.0-5.20)* | - |
| Lack of friend or confidant | No  Yes | REF  0.90 (0.60-1.20) | - |
| Husband’s reaction to dowry | Did not bring/did not care  Satisfied  Unsatisfied | REF  1.0 (0.5–2.0)  13.3 (3.8–46.0)* | - |
| In-law’s reaction to dowry | Did not bring/did not care  Satisfied  Unsatisfied | REF  1.0 (0.5–2.0)  17.7 (4.5–69.3)* | REF  0.90 (0.5-2.0)  11.2 (2.4–52.9)* |

REF=reference group

OR=Odds Ratio

AOR=Adjusted odds ratio

*Statistically significant at 0.05 significance level
