# Supplementary material for: Social determinants of antenatal depression and anxiety among women in South Asia: A systematic review & meta-analysis
Source: PLoS One. 2022 Feb 9;17(2):e0263760. doi: 10.1371/journal.pone.0263760 (PMC8827460; doi:10.1371/journal.pone.0263760)
Supplement: S5 Table — (DOCX) [file pone.0263760.s008.docx]

**S5 Table** Factors included in the included studies’ adjusted models

| **Author & Study year** | **Factors included in adjusted model** |
| --- | --- |
| Ajinkya et al, 2013 | No adjusted model |
| Ali et al, 2012 | Maternal age, total live births, adverse pregnancy outcome, respondent’s role in household decision making, domestic violence |
| Ayaz et al, 2019 | No adjusted model |
| Ayyub et al, 2018 | Maternal age, death of close relative (within 1 year), intimate partner violence, food insecurity |
| Babu et al, 2018 | Maternal age, gestational age, total income, respondent education, occupation, and husband’s occupation |
| Bavle et al, 2016 | No adjusted model |
| Dahiya et al, 2020 | No adjusted model |
| Din et al, 2016 | Maternal age, husband support in pregnancy, monthly family income, family size, stressful life events, lack of confidence in day-to-day life, domestic violence, pregnancy-related concerns, family/social problems (with in-laws), history of depression, home ownership status, family type, gravidity |
| Gausia et al, 2009 | Education 0–5 years schooling, wanted son, unwanted pregnancy, no help received from Mother-in-law, history of mental illness, no help received from Husband, poor relationship with mother-in-law, beaten by husband during pregnancy and before |
| George et al, 2016 | financial difficulties, violence by intimate partner, presence of complications in the cur- rent pregnancy, history of miscarriage/still birth, history of marital conflict, difficult relationship with mother-in-law, being in a non-arranged marriage, current trimester of pregnancy, pressure to have a male child |
| Ghaffar et al, 2017 | Maternal age, education, occupation, monthly income, locality, ethnic group, number of children |
| Goyal et al, 2020 | No adjusted model |
| Gul et al, 2017 | No adjusted model |
| Hegde et al, 2013 | No adjusted model |
| Humayun et al, 2013 | No adjusted model |
| Imran et al, 2010 | No adjusted model |
| Jafri et al, 2017 | No adjusted model |
| Jamal et al, 2018 | Gestational age, gravidity, parity, past history of abortion/miscarriage, history of C section, household monthly income, fear associated with childbirth, unplanned pregnancy, separation from the husband, childhood traumatic experiences, other stressful life events |
| Karmaliani et al, 2009 | Maternal age, formal education, informal education, employment, husband’s employment, property index, number of previous pregnancies, wanted this pregnancy domestic violence |
| Maselko et al, 2018 | Woman’s age and number of living children |
| Mir et al, 2012 | Age, the participants’ educational level, family system, grand-multiparity, unplanned pregnancy, marital satisfaction, loss of parents, verbal/physical abuse, and substance use |
| Nasreen et al, 2011 | Age, literacy, poor household economic status, involve in income-generating activities, relationship with husband, practical support, forced sex, physical violence, parity, history of child death, planned pregnancy, previous history of mental illness |
| Nath et al, 2019 | Social support, marital discord, spouse violence |
| Niaz et al, 2004 | No adjusted model |
| Rabia et al, 2017 | No adjusted model |
| Rahman et al, 2003 | Earning member of the family (usually the husband) being made redundant, financial difficulties, housing problems, serious argument or relationship difficulties with a significant member of the extended family, serious marital problems, presence of two or more children under the age of seven, or two or more girl-children, support from extended family, practice of traditional ‘chilla’ ritual, Support by family members with routine child-care, presence of the infant’s grandmother, husbands literacy, husbands employment |
| Sabir et al, 2019 | No adjusted model |
| Safi et al, 2013 | No adjusted model |
| Sheeba et al, 2019 | number of pregnancies (gravida), unplanned pregnancy, low social support, presence of marital discord, domestic violence, catastrophic events |
| Shehroz et al, 2019 | No adjusted model |
| Shidhaye et al, 2017 | Woman’s education, caste, age at marriage, intimate partner violence, husband’s reaction to dowry, in-laws’ reaction to dowry, problems in relationship with husband, problems in relationship with in-laws, trimester of pregnancy |
| Srinivasan et al, 2015 | No adjusted model |
| Surkan et al, 2018 | Maternal education, maternal age, parity, maternal literacy, maternal employment, religion, maternal mid-upper arm circumference in the first trimester, anemia in first trimester (yes/no), vitamin supplementation group (vitamin A, beta-carotene, placebo), geographic sector |
| Zia et al, 2018 | No adjusted model |
